# UK Medical Cannabis Registry: A Two‐Year Prospective Observational Cohort Study of Clinical Outcomes in Insomnia

**DOI:** 10.1002/npr2.70180

**Published:** 2026-10-26

**Authors:** Aran Koye, Simon Erridge, Evonne Clarke, Katy McLachlan, Ross Coomber, James J. Rucker, Mark W. Weatherall, Mikael H. Sodergren

**Affiliations:** ^1^ Medical Cannabis Research Group Imperial College London London UK; ^2^ Curaleaf Clinic London UK; ^3^ St. George's University Hospitals NHS Trust London UK; ^4^ Department of Psychological Medicine King's College London London UK; ^5^ South London & Maudsley NHS Foundation Trust London UK

**Keywords:** cannabidiol, cannabis, insomnia, sleep, tetrahydrocannabinol

## Abstract

**Background:**

Insomnia disorder affects approximately 10% of people. The persistence of insomnia, despite treatment, remains high. Cannabis‐based medicinal products (CBMPs) have been proposed as a potential treatment for insomnia. This study aims to use data from the UK Medical Cannabis Registry to analyze changes in self‐reported sleep quality in patients prescribed CBMPs for insomnia.

**Methods:**

A prospective cohort study of patients treated with CBMPs for insomnia was completed. The primary outcome was the Single‐Item Sleep Quality Scale (SQS). This was assessed at baseline and at 1, 3, 6, 12, 18, and 24 months from baseline. Secondary outcomes included change in anxiety and general health‐related quality of life. *p*‐values < 0.050 were considered statistically significant.

**Results:**

Two hundred and eleven participants met the inclusion criteria. Over half (*n* = 126; 59.72%) were users of cannabis at baseline. At the primary endpoint of 24 months, mean SQS improved from 2.46 ± 2.10 (mean ± standard deviation (SD)) at baseline to 5.14 ± 2.75 (mean difference +2.68; 95% confidence interval (CI) 2.23–3.14; *p* < 0.001), with improvements observed at all timepoints. SQS values of 0 (odds ratio (OR) 15.49; 95% CI 3.87–65.19; *p* < 0.001) and 1–3 (OR 4.90; 95% CI 1.87–13.80; *p* = 0.002) at baseline and prescription of dried flower CBMPs (OR 4.99; 95% CI 1.27–21.39; *p* = 0.025) were associated with clinically significant improvements in sleep quality at 24 months. Seventeen participants (8.06%) reported adverse events. Participants aged over 60 were more likely to report an adverse event (OR 19.08; 95% CI 1.52–535.75; *p* = 0.036).

**Conclusion:**

Initiation of CBMPs was associated with improvements in self‐reported sleep quality across 2 years, with the largest associations observed in those with the most severe impairment. As an uncontrolled, observational study of a self‐selected and predominantly cannabis‐experienced cohort, placebo effects, regression to the mean, and selection bias cannot be excluded.

## Abstract 2

Insomnia affects around one in ten adults. In this 2‐year UK Medical Cannabis Registry cohort study of 211 adults with insomnia, treatment with cannabis‐based medicinal products was associated with a sustained improvement in self‐reported sleep quality across 24 months. Adverse events were uncommon but more frequently reported by adults over 60.

## Graphical abstract

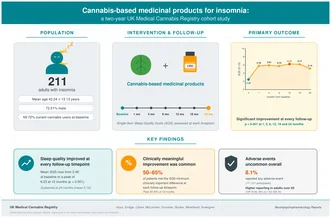

## 1 Introduction

Insomnia is a debilitating sleep disorder that affects around 10% of people globally [1, 2, 3, 4, 5]. It is defined by persistent difficulties in initiating sleep, maintaining sleep, or experiencing poor sleep quality despite adequate opportunity and appropriate sleeping conditions on at least 3 nights per week for at least 3 months, with associated daytime impairment [6]. Common sleep disturbances associated with insomnia include problems with sleep initiation, sleep maintenance, and early morning wakening [7]. These impairments lead to decreased self‐perceived energy levels, diminished biopsychosocial functioning, and reduced health‐related quality of life (HRQoL) [8, 9].

The societal burden of insomnia is profound, with both indirect and direct impacts on the economy. Insomnia carries an economic burden of approximately £34 billion GBP in the UK alone, using 2019 estimates, due to reduced workplace activity, heightened accident risk, and increased healthcare use [10, 11]. The burden of insomnia is exacerbated by high rates of comorbidity with other chronic illnesses [12, 13], compounding its impact on healthcare systems and individuals.

Hypnotic medications are commonly prescribed for insomnia treatment, notably Z‐drugs and benzodiazepines [14, 15, 16]. However, long‐term use of these agents is associated with risks, including falls and fractures in patients older than 65, and dependency [17, 18]. Consequently, the National Institute for Health and Care Excellence guidelines advise against the use of hypnotics in elderly populations and in pregnant or breastfeeding individuals [19]. While melatonin has become popular as an alternative treatment option, current research does not provide sufficient evidence to support its efficacy in managing insomnia [20]. Dual orexin receptor antagonists (DORAs) are novel therapies designed for insomnia. Daridorexant is currently the only licensed DORA in the UK; however, there is limited evidence of its long‐term safety and efficacy [21]. This highlights the continued need for safe and effective evidence‐based therapeutic options. Management of insomnia has also proven difficult as many patients are resistant to current therapies [4, 22]. A longitudinal study by Morin and colleagues suggests that approximately 6 in 10 individuals with insomnia disorder have ongoing symptoms after 5 years [23].

A neurobiological signaling network known as the endocannabinoid system (ECS) has shown promise as a potential therapeutic target due to emerging evidence on its regulatory role in the sleep–wake cycle and effects on sleep architecture [24]. The ECS is composed of primary cannabinoid receptors, cannabinoid receptor 1 (CB1) and cannabinoid receptor 2 (CB2), their respective ligands and enzymes [25]. CB1 is the most abundant G protein‐coupled receptor in the brain [26]. Activation of the CB1 receptor via endogenous CB1 agonists such as anandamide promotes rapid eye movement (REM) and non‐REM sleep, which are both disrupted in insomnia patients [24, 27].

Cannabis‐based medicinal products (CBMPs) have been explored in recent years as a potential treatment for a variety of conditions including insomnia. The cannabis plant is comprised of over 100 phytocannabinoids, most notably (−)trans‐Δ9‐tetrahydrocannabinol (THC) and cannabidiol (CBD) [28]. THC acts as a partial agonist at CB1 receptors [29]. CBD displays limited direct binding to cannabinoid receptors. It indirectly modulates the activity of CB1 by reducing anandamide degradation, thus increasing CB1 activation [30, 31]. Research suggests that cannabinoids may reset disruptions to the sleep–wake cycle while reducing sleep latency and prolonging sleep duration [32]. Moreover, the broader ECS network, the endocannabinoidome, includes several other receptors which influence the sleep–wake cycle such as peroxisome proliferator activated receptors (PPARs) and transient receptor potential (TRP) channels [33, 34]. Activation of PPARs, particularly PPARα, has been linked to increased wakefulness and activity in mice during food shortages by promoting ketone production, helping to maintain energy levels and prevent excessive sleep [35]. TRP channels serve as modulators of the sleep–wake cycle in humans by regulating neuronal and metabolic activity and influencing circadian gene expression in response to environmental changes [36].

There is emerging evidence on the effects of CBMPs on sleep, particularly in patients with psychiatric conditions such as anxiety and post‐traumatic stress disorder (PTSD) [37, 38]. A recent narrative review also highlights the benefits and safety of CBMP use [39]. It is important to note that different cannabinoids such as THC, CBD, and cannabinol (CBN) exert distinct clinical effects [40, 41]. The therapeutic outcomes of CBMPs can vary greatly depending on their specific cannabinoid composition and dosing. ZTL‐101, a CBMP containing 20 mg/mL of THC, 1 mg/mL of CBD, and 2 mg/mL of CBN, has shown promise as a safe and effective insomnia treatment [40]. This formula leverages sedative effects associated with THC and CBN [40]. While these findings are encouraging, data from this study are limited due to short follow‐up periods, small patient numbers, and assessment of sleep quality via actigraphy rather than polysomnography. There is still a need for more robust data to truly develop our understanding of cannabinoid use in the treatment of various conditions.

Current research on CBMP use for insomnia has been limited due to most study designs being observational in nature, allowing for potential bias and confounders [42]. Current research also lacks consistent terminology and measurement tools to assess the effects of CBMP use [43]. Other limitations include lack of long‐term clinical studies, small sample size, and large variations in outcomes in current studies [44, 45, 46]. Long‐term use of CBMPs may also carry a risk of developing a tolerance to therapeutic effects [47]. There is also concern about CBMPs containing THC negatively impacting the quality of REM sleep [48, 49]. Existing studies are limited by narrow focus, with little information on patient and treatment‐related variables that may have an influence on the incidence of these side effects [43].

This cohort study aims to use data from the UK Medical Cannabis Registry (UKMCR) to assess changes in self‐reported sleep quality in patients prescribed CBMPs for the primary indication of insomnia. Secondary aims include assessing the incidence of adverse events (AEs) and investigating factors found to be associated with a clinically significant improvement in insomnia outcomes.

## 2 Methods

### 2.1 Study Design

This is a two‐year prospective observational cohort study of patients prescribed CBMPs for the primary indication of insomnia, drawn from the UKMCR. Patient‐reported outcome measures (PROMs) were collected from patients at baseline and months 1, 3, 6, 12, 18, and 24 via online surveys. AEs were also reported throughout the study either contemporaneously, following prompting on completion of PROMs, or by clinicians during consultations. Data extraction occurred on 6th January 2025. The UKMCR has received ethical approval from the Central Bristol Research Ethics Committee (Reference 22/SW/0145).

### 2.2 Settings and Participants

The UKMCR is privately owned by Curaleaf Clinic and was established in December 2019. It is currently the largest database of pseudonymised information on patients in the UK who are prescribed CBMPs. Written consent was collected from each patient before consecutive enrolment into the study. The registry collects longitudinal data from clinical encounters through a custom electronic platform which participants have previously reported as user‐friendly for submitting PROMs [50].

Participants were identified according to the following criteria:

– Inclusion: Patients who are prescribed CBMPs for the primary indication of insomnia.
– Exclusion: Those who did not complete any baseline PROMs and those who had enrolled fewer than 2 years prior to data extraction.

Demographic data were collected during the initial clinical encounter, including age, sex, occupation, weight, height, body mass index (BMI), indication for CBMP treatment, comorbidities, drug and alcohol data, and previous cannabis use prior to commencing treatment.

All patients were prescribed CBMPs in line with current guidance in the UK [51]. Patients may be prescribed unlicensed CBMPs if licensed treatments have failed to show a beneficial effect or are not suitable for use by the patient for the presenting indication, in this case insomnia [51]. All medications are manufactured in accordance with Good Manufacturing Practice [51].

The products available for prescription were CBMPs in the form of either capsules, ointments, pastilles, vape cartridges, oils, or dried flowers. Capsules and pastilles are administered orally. Oils are administered orally or sublingually, and ointments topically. Dried flowers and vape cartridges are vaporized and inhaled.

### 2.3 Outcome Measures

The primary outcome variable was the Single‐Item Sleep Quality Scale (SQS). The primary endpoint was the change in SQS from baseline to 24 months. Changes in SQS from baseline to 1, 3, 6, 12, and 18 months were assessed as secondary endpoints. Other secondary outcome variables were the Generalized Anxiety Disorder‐7 (GAD7) score, the EuroQol 5‐Dimension 5‐Level (EQ‐5D‐5L) index value, and the Patient Global Impression of Change (PGIC) questionnaire. All outcome variables were assessed at baseline and at 1, 3, 6, 12, 18, and 24 months after the first prescription, except the PGIC questionnaire which was not collected at baseline. Patient‐reported outcome measures (PROMs) were collected through a custom electronic platform that participants have previously reported as user‐friendly for this purpose.

AEs may be recorded on the electronic platform by clinicians during routine clinical encounters or by patients either contemporaneously or during completion of PROMs questionnaires. These AEs were defined according to the Common Terminology Criteria for Adverse Events version 4.0.

### 2.4 SQS

For the SQS, participants are asked to rate their overall sleep quality on a scale of 0–10, where 0 is “terrible” and 10 is “excellent” sleep quality [52]. Previous studies have shown that a change in single‐item SQS of 2.6 or more from baseline constitutes a clinically significant result [53]. The SQS has shown strong internal and external reliability, with an internal consistency of 0.92 (Cronbach's *α* score) and a test–retest score of 0.81 (intraclass correlation coefficient) [52]. When compared to other sleep quality measures such as the Pittsburgh Sleep Quality Index and the Morning Questionnaire‐Insomnia, the single item SQS shows strong correlations, supporting the validity of the scale [53].

### 2.5 GAD7

The GAD7 questionnaire is made up of 7 questions, each assigned a score of 0–3 [54]. The self‐reported questionnaire assesses severity of generalized anxiety disorder with higher scores indicating more severe symptoms associated with anxiety [54]. A clinically significant change in GAD7 score is a change of 4 points [55]. The questionnaire has shown internal reliability with a consistent Cronbach's α score of approximately 0.90 [56, 57], and external reliability with an interclass correlation coefficient of 0.83 [54]. Evidence has also supported the validity of the GAD7 score as a tool to measure anxiety within the general population [56, 57].

### 2.6 EQ‐5D‐5L

The EQ‐5D‐5L is a self‐reported measure of HRQoL that provides users with an index score ranging from −0.59 to 1, where 1 represents the highest HRQoL and negative scores represent a health status that is perceived to be worse than dead [58]. The tool consists of 5 dimensions including mobility, self‐care, usual activities, pain/discomfort, and anxiety/depression. For each dimension, users report the level of problems they experience from a range of 5 answers, and the index value is subsequently calculated [58]. There is currently no universally accepted clinically significant change of the EQ‐5D‐5L index value across all countries and conditions. The EQ‐5D‐5L is a valid and reliable measure with statistically significant test–retest and Cronbach's α scores of around 0.9 and 0.8 respectively [59, 60, 61].

### 2.7 PGIC

The PGIC is another self‐reported measure of HRQoL that is scored on a 7‐point scale [62]. It is considered a “gold standard” measure of clinically significant change [63]. The score reflects the user's belief about the efficacy of their treatment [62]. Patients rate the change in their health status as one of 7 levels ranging from “no change or worsening” to “a great deal better, and a considerable improvement” [62].

### 2.8 Missing Data

Missing PROM values were addressed using multiple imputation by chained equations (mice package in R), generating five imputed datasets and pooling estimates across imputations using Rubin's rules. Only PROMs were included in the imputation model, ensuring that missing values were imputed from participants with similar outcome trajectories.

### 2.9 Statistical Analysis

Descriptive statistics were used to report demographic data, CBMP prescriptions, medical history, and AEs. Distributional assumptions for continuous variables were assessed using the Shapiro–Wilk test together with visual inspection of histograms and Q–Q plots. Continuous variables are presented as both mean ± standard deviation (SD) and median with interquartile range (IQR). The PROM values were analyzed using a repeated‐measures analysis of variance (ANOVA) in accordance with the Central Limit Theorem. The repeated‐measures ANOVA assesses differences between paired values from the same patients across time, accounting for the within‐subject correlation of repeated measurements under a compound‐symmetry assumption. Where the omnibus repeated‐measures ANOVA reached statistical significance, post hoc paired comparisons were performed between each timepoint. The Bonferroni correction was applied within each ANOVA family with 21 comparisons. For the primary outcome, the mean and the mean difference from baseline are reported as point estimates with their corresponding SD and 95% confidence interval (CI). AEs were summarized using frequency counts and percentages. Univariable and multivariable logistic regression models were performed to evaluate any associations between demographic and clinical variables and the occurrence of a minimum clinically important difference (MCID) value in SQS and GAD7, and any positive change in EQ‐5D‐5L. Logistic regressions of these values were used in preference to linear regression because these scales are not truly continuous and are rather ordinal scales, which would otherwise violate the assumptions of constant variance. Univariable and multivariable logistic regressions were also performed to assess the association between variables and the reporting of one or more AEs. Results of logistic regression were presented as the odds ratio (OR) with the corresponding 95% CI. The Bonferroni correction was applied within each logistic regression. *p* < 0.050 was considered statistically significant. All analyses were conducted using RStudio in the programming language R (version 4.4.3; R Foundation for Statistical Computing, Vienna, Austria).

## 3 Results

### 3.1 Patient Data

On the date of data extraction, 34 563 patients had enrolled on the UKMCR (Figure 1). 3055 (8.84%) were excluded for not completing baseline PROMs, leaving 31 508 (91.16%) patients. Of these, 8945 (25.88%) had enrolled > 2 years before extraction; 211 (0.61%) with insomnia as the primary indication were included.

**FIGURE 1:**
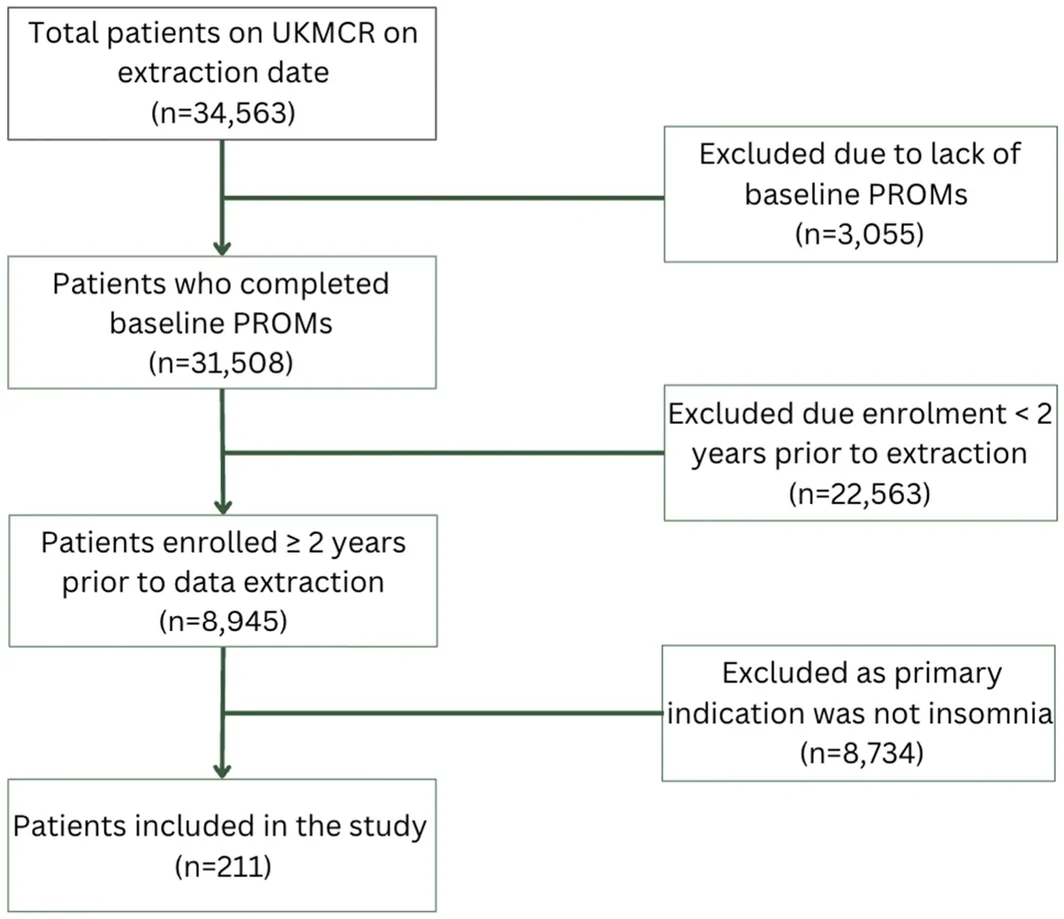
Flowchart showing patient selection from the UK Medical Cannabis Registry (UKMCR). Data extraction occurred on 6th January 2025. Two hundred and eleven patients met the final selection criteria and were included in the study. PROMs, patient‐reported outcome measures.

### 3.2 Demographics

The mean age of participants was 42.24 ± 13.13 years and 72.51% (*n* = 153) of participants were male (Table **1**). The median Charlson Comorbidity Index was 0.00 (IQR: 0.00–5.00). A score of 0 was calculated for 142 (67.30%) participants and a score of 1 or greater was calculated for 69 (32.70%) participants. Depression/anxiety was the most common comorbidity (47.39%; *n* = 100; Table S1).

**TABLE 1:** Baseline demographics and patient characteristics (*n* = 211).

Characteristic	*n* (%)/mean ± SD/median (IQR)
Age (years)	42.24 ± 13.13; 40.00 (32.00–50.00)
BMI^a^	26.77 ± 5.57; 26.30 (22.80–29.40)
Sex	
Male	153 (72.51)
Female	58 (27.49)
Occupation^b^	
Clerical support workers	5 (2.37)
Craft and related trade workers	13 (6.16)
Elementary occupations	7 (3.32)
Managers	13 (6.16)
Plant and machine operators, and assemblers	1 (0.47)
Professional	41 (19.43)
Services and sales workers	11 (5.21)
Skilled agricultural forestry and fishery workers	6 (2.84)
Technicians and associate professionals	21 (9.95)
Other occupations	42 (19.91)
Retired	2 (0.95)
Unemployed	39 (18.48)
Charlson Comorbidity Index Score	1.73 ± 2.64; 0.00 (0.00–5.00)
Score 0	142 (67.30)
Score ≥ 1^c^	69 (32.70)

*Note:* Baseline demographics of participants. Continuous variables are reported as mean ± SD and median (IQR); categorical variables as *n* (%). Body mass index (BMI) is calculated by dividing each patient's weight in kilograms by their height in meters squared and is measured in kg/m^2^.

Abbreviations: IQR, interquartile range; SD, standard deviation.

^a^ BMI missing data = 8/211 (3.79%).

^b^ Occupation missing data = 10/211 (4.74%). All other variables in table had complete data.

^c^ Membership of specific Charlson comorbidity categories ≥ 1 contain fewer than five individuals, with some containing as few as one individual and may produce inadvertent reidentification so have been aggregated into one category.

The most common secondary indications for treatment were anxiety (*n* = 38; 18.01%), chronic non‐cancer pain (*n* = 12; 5.69%), and depression (*n* = 7; 3.32%; Table S2). The most common tertiary indications were depression (*n* = 8; 3.79%), anxiety (*n* = 2; 0.95%), and attention deficit hyperactivity disorder (*n* = 2; 0.95%).

The median weekly alcohol intake at baseline was 1.50 [0.00–7.50] units (Table 2). The proportion of current smokers at baseline was 29.38% (*n* = 62). A further 45.50% (*n* = 96) of participants were ex‐smokers. The remaining 53 participants (25.12%) had never smoked. The lifetime tobacco exposure for both current and ex‐smokers was 10.00 [3.00–20.00] pack years.

**TABLE 2:** Baseline alcohol, tobacco, and cannabis history (*n* = 211).

	*n* (%)/mean ± SD; median (IQR)
Weekly alcohol consumption in units	6.69 ± 13.70; 1.50 (0.00–7.50)
Smoking status	
Never smoked	53 (25.12)
Ex‐smoker	96 (45.50)
Current smoker	62 (29.38)
Smoking pack years (current/ex‐smokers)	14.37 ± 17.37; 10.00 (3.00–20.00)
Recreational cannabis use	
Never used	40 (18.96)
Ex‐user	45 (21.33)
Current user	126 (59.72)
Cannabis gram years (current/ex‐users)	15.32 ± 20.92; 7.00 (2.00–20.00)

*Note:* Reports use of alcohol, tobacco, and cannabis by participants at baseline. Continuous variables are reported as mean ± SD and median (IQR); categorical variables as *n* (%). All variables in table had complete data.

Abbreviations: IQR, interquartile range; SD, standard deviation.

The proportion of participants who were consumers of cannabis at baseline prior to starting treatment was 59.72% (*n* = 126). Of this group, the lifetime cannabis exposure was 10.00 [3.00–20.00] gram years. A further 21.33% (*n* = 45) were ex‐users of cannabis and the lifetime cannabis exposure for this group was 4.00 [1.00–15.00] gram years.

### 3.3 CBMPs

At baseline, 40.76% of participants (*n* = 86) were prescribed CBMPs in the form of dried flower/flos only, 29.86% (*n* = 63) were prescribed oil‐based CBMPs only, and 29.38% (*n* = 62) were prescribed a combination of both (Table 3). At 24 months 41.23% of participants (*n* = 87) were prescribed CBMPs in the form of dried flower/flos only, 17.06% (*n* = 36) were prescribed oil‐based CBMPs only, and 32.23% (*n* = 68) were prescribed a combination of both. At 2 years, due to the introduction of other formats, there were an increasing number (*n* = 20; 9.48%) who were prescribed other combinations. The median THC dose was 20.00 (2.00–21.86) mg/day at baseline and 143.00 (110.00–247.50) mg/day at 24 months. The median CBD dose was 1.50 (1.00–20.00) mg/day and 13.75 (6.18–48.40) mg/day at baseline and 24 months.

**TABLE 3:** Cannabis‐based medicinal product prescriptions at baseline, 1, 3, 6, 12, 18, and 24 months (*n* = 211).

CBMP preparation	*n* (%)						
	Baseline	1 month	3 months	6 months	12 months	18 months	24 months
Dried flower/Flos only	86 (40.76)	77 (36.49)	74 (35.07)	81 (38.39)	88 (41.71)	83 (39.34)	87 (41.23)
Oil only	63 (29.86)	57 (27.01)	48 (22.75)	37 (17.54)	35 (16.59)	36 (17.06)	36 (17.06)
Dried flower/Flos and oil	62 (29.38)	75 (35.55)	86 (40.76)	89 (42.18)	84 (39.81)	81 (38.39)	68 (32.23)
Other^a^	0 (0.00)	2 (0.95)	3 (1.42)	4 (1.90)	4 (1.90)	11 (5.21)	20 (9.48)

Cannabinoid dose	Median (IQR)						
	Baseline	1 month	3 months	6 months	12 months	18 months	24 months
CBD dose (mg/day)	1.50 (1.00–20.00)	11.00 (5.50–25.50)	11.00 (5.50–27.50)	11.55 (5.50–27.50)	11.55 (5.93–38.00)	15.41 (7.80–49.50)	13.75 (6.18–48.40)
THC dose (mg/day)	20.00 (2.00–21.86)	110.00 (12.80–121.00)	120.50 (88.00–208.25)	121.00 (110.00–280.88)	137.50 (110.00–247.50)	143.00 (110.00–243.00)	143.00 (110.00–247.50)

*Note:* Dried flower preparations were vaporized, while oil preparations were used sublingually. Data is expressed as number (%).

Abbreviations: CBD, cannabidiol; THC, (−)trans‐Δ^9^‐tetrahydrocannabinol.

^a^ Other CBMP combinations combined to prevent inadvertent reidentification. All variables in table had complete data.

### 3.4 PROMs

Figure 2 presents a detailed summary of PROM results. Further detailed breakdowns (including MCID values) can be found in Table S3. In repeated measures ANOVA, there was a significant effect of time on the SQS, GAD7, and EQ‐5D‐5L index value (*p* < 0.001).

**FIGURE 2:**
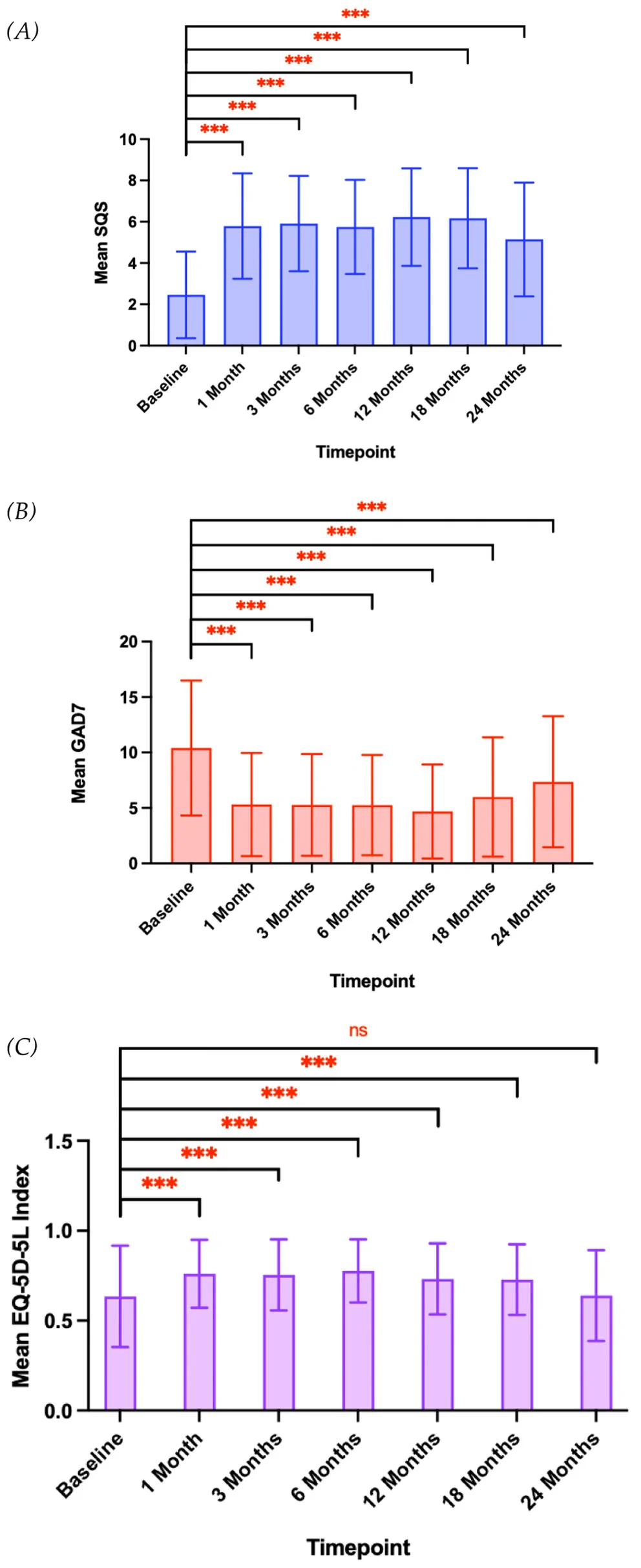
Mean patient reported outcome measures (PROMs) at baseline, 1, 3, 6, 12, 18, and 24 months (*n* = 211). (A) Mean ± standard deviation Single‐Item Sleep Quality Scale (SQS) score at each time point. Every timepoint had a higher mean SQS score compared to baseline (*p* < 0.001). (B) Mean ± standard deviation Generalized Anxiety Disorder‐7 (GAD7) score at each time point. Every timepoint had a lower mean GAD7 score compared to baseline (*p* < 0.001). (C) Mean ± standard deviation EuroQol 5‐dimension 5‐level (EQ‐5D‐5L) index value at each time point. Every timepoint had a higher mean EQ‐5D‐5L index value compared to baseline (*p* < 0.001), except 24 months (*p* > 0.999). Missing PROMs at 1 (*n* = 12; 5.69%), 3 (*n* = 38; 18.01%), 6 (*n* = 61; 28.91%), 12 (*n* = 94; 44.55%), 18 (*n* = 116; 54.98%), and 24 months (*n* = 135; 63.98%) were handled with multiple imputation. *p < 0.050; **p < 0.010; ***p < 0.001; ns, not significant.

Relative to baseline (mean ± SD: 2.46 ± 2.10, 95% CI 2.17–2.74), SQS improved at every follow‐up timepoint (*p* < 0.001). At the primary endpoint of 24 months, the mean was 5.14 ± 2.75 (95% CI 4.77–5.52), corresponding to a mean difference from baseline of +2.68 ± 3.37 (95% CI 2.23–3.14; *p* < 0.001). The mean SQS values and mean differences from baseline at secondary timepoints were 5.79 ± 2.55 (95% CI 5.45–6.14; +3.33 ± 2.72, 95% CI 2.96–3.70) at 1 month, 5.91 ± 2.31 (95% CI 5.59–6.22; +3.45 ± 2.82, 95% CI 3.06–3.83) at 3 months, 5.75 ± 2.28 (95% CI 5.44–6.06; +3.29 ± 2.83, 95% CI 2.90–3.67) at 6 months, 6.23 ± 2.36 (95% CI 5.91–6.55; +3.77 ± 2.95, 95% CI 3.37–4.17) at 12 months, and 6.17 ± 2.42 (95% CI 5.84–6.50; +3.71 ± 3.01, 95% CI 3.30–4.12) at 18 months (all *p* < 0.001 after Bonferroni correction).

Univariable logistic regression analysis indicated that participants with baseline GAD7 ≥ 15 were more likely to report a clinically significant improvement in SQS (OR = 3.45; 95% CI 1.56–7.95; *p* = 0.003). The univariable outcomes are detailed in full in Table S4.

In multivariable logistic regression analysis, the association with baseline GAD7 ≥ 15 was attenuated and no longer statistically significant (OR = 2.07; 95% CI 0.70–6.31; *p* = 0.193). Multivariable analysis also revealed that patients prescribed dried flower alone were more likely to experience a clinically significant improvement (OR = 4.99; 95% CI 1.27–21.39; *p* = 0.025). Greater baseline sleep impairment was also associated with reporting the MCID at 24 months (baseline SQS 1–3: OR = 4.90; 95% CI 1.87–13.80; *p* = 0.002; baseline SQS 0: OR = 15.49; 95% CI 3.87–65.19; *p* < 0.001; reference: SQS 4–10). A detailed summary of SQS‐specific multivariable data is shown in Table 4.

**TABLE 4:** Multivariable logistic regression for the association between patient and treatment variables and a minimal clinically important difference (MCID) in the Single‐Item Sleep Quality Scale (SQS).

Category	*n*	Adjusted odds ratio (95% CI)	*p*
Age (years)			
≤ 30	43	—	—
31–40	59	2.06 (0.76–5.58)	0.143
41–50	52	0.86 (0.32–2.29)	0.766
51–60	29	3.11 (0.92–11.14)	0.072
> 60	20	0.51 (0.12–2.03)	0.341
Sex			
Male	149	—	—
Female	54	1.18 (0.52–2.67)	0.684
BMI (kg/m^**2**^)			
20–24.99	69	—	—
< 20	15	1.23 (0.32–4.97)	0.767
25–29.99	72	0.70 (0.30–1.68)	0.402
30–34.99	33	0.40 (0.14–1.08)	0.076
≥ 35	14	0.75 (0.18–3.19)	0.692
Cannabis status			
Never used	37	—	—
Current user	122	0.52 (0.17–1.53)	0.237
Ex‐user	44	0.82 (0.24–2.79)	0.754
CBMP route of administration at 24 months			
Oil	33	—	—
Dried flower and oil	64	3.52 (0.84–15.76)	0.090
Dried flower	86	4.99 (1.27–21.39)	0.025
Other	20	1.46 (0.32–6.89)	0.629
Total CBD dosage at 24 months			
Minimum to Q1	51	—	—
Q1 to Median	51	1.77 (0.59–5.55)	0.313
Median to Q3	49	2.10 (0.64–7.22)	0.229
Q3 to Maximum	52	1.89 (0.60–6.12)	0.279
Total THC dosage at 24 months			
Minimum to Q1	64	—	—
Q1 to Median	37	0.26 (0.07–0.92)	0.042
Median to Q3	50	0.16 (0.04–0.56)	0.005
Q3 to Maximum	52	0.47 (0.12–1.72)	0.262
Baseline SQS			
4–10	41	—	—
1–3	124	4.90 (1.87–13.80)	0.002
0	38	15.49 (3.87–65.19)	< 0.001
Baseline GAD7			
< 5	43	—	—
5–9	49	1.08 (0.38–3.07)	0.884
10–14	54	1.15 (0.40–3.27)	0.797
≥ 15	57	2.07 (0.70–6.31)	0.193

Abbreviations: BMI, body mass index; CBD, cannabidiol; CBMP, cannabis‐based medicinal product; CI, confidence interval; GAD7, Generalized Anxiety Disorder‐7; OR, odds ratio; SQS, Single‐Item Sleep Quality Scale; THC, (−)trans‐Δ9‐tetrahydrocannabinol.

The mean GAD7 score was also improved at all timepoints against baseline (10.41 ± 6.08; *p* < 0.001). At 24 months, 48.82% (*n* = 103) of patients reported a clinically significant improvement and the mean GAD7 score was 7.36 ± 5.91 (*p* < 0.001).

Univariable analysis showed that patients with a baseline SQS of 0 and 1–3 had higher odds of reporting the MCID in GAD7 (OR = 5.23; 95% CI 2.08–13.96; *p* < 0.001, and OR = 2.95; 95% CI 1.40–6.61; *p* = 0.006 respectively; Table S5). However, these associations were not maintained in multivariable analysis (baseline SQS 0: OR = 1.55; 95% CI 0.42–5.90; *p* = 0.513; baseline SQS 1–3: OR = 1.08; 95% CI 0.36–3.25; *p* = 0.896; Table S6).

Multivariable analysis suggests that participants who were prescribed a combination of dried flower and oil CBMPs were more likely to report a MCID in GAD7 (OR = 8.44; 95% CI 1.33–66.38; *p* = 0.030; Table S6). Patients who used higher doses of THC were less likely to display a clinically significant improvement in GAD7 score (THC Q1 to Median: OR = 0.09, 95% CI 0.01–0.45, *p* = 0.006; Median to Q3: OR = 0.08, 95% CI 0.01–0.40, *p* = 0.004; Q3 to Max: OR = 0.13, 95% CI 0.02–0.68, *p* = 0.023).

The mean EQ‐5D‐5L index value was improved versus the baseline mean of 0.63 (±0.28) at all timepoints (*p* < 0.001), except 24 months (0.64 ± 0.25; *p* > 0.999).

Univariable analysis showed that patients with baseline GAD7 scores of 10–14 (OR = 3.67; 95% CI 1.60–8.79; *p* = 0.003) and ≥ 15 (OR = 5.50; 95% CI 2.40–13.29; *p* < 0.001) were more likely to report an improvement in EQ‐5D‐5L index value (Table S7). This was also observed on multivariable analysis (GAD7 10–14: OR = 3.24; 95% CI 1.23–8.98; *p* = 0.020; GAD7 ≥ 15: OR = 4.51; 95% CI 1.64–13.08; *p* = 0.004; Table S8).

The PGIC remained consistent with a median of 6 across all measured timepoints (Table S9).

### 3.5 Adverse Events

Seventeen (8.06%) participants reported one or more AE. The most common adverse events were insomnia (*n* = 13; 6.16%), fatigue (*n* = 12; 5.69%), and dry mouth (*n* = 10; 4.74%; Table 5). No participants experienced life‐threatening AEs.

**TABLE 5:** Adverse events reported by participants.

Adverse event	*n* (%)	Mild	Moderate	Severe
Insomnia	13 (6.16)	0	8	5
Fatigue	12 (5.69)	5	7	0
Other (single occurrence)	11 (5.21)	4	5	2
Dry mouth	10 (4.74)	8	2	0
Somnolence	9 (4.27)	0	8	1
Dizziness	8 (3.79)	4	4	0
Lethargy	8 (3.79)	6	2	0
Headache	7 (3.32)	6	1	0
Vertigo	7 (3.32)	5	2	0
Concentration impairment	5 (2.37)	3	1	1
Dyspepsia	5 (2.37)	2	3	0
Nausea	4 (1.90)	4	0	0
Amnesia	3 (1.42)	1	1	1
Constipation	3 (1.42)	3	0	0
Delirium	3 (1.42)	1	1	1
Pharyngitis	3 (1.42)	1	2	0
Ataxia	2 (0.95)	1	1	0
Cognitive disturbance	2 (0.95)	1	1	0
Confusion	2 (0.95)	1	1	0
Cough	2 (0.95)	1	1	0
Dysgeusia	2 (0.95)	0	2	0
Tremor	2 (0.95)	2	0	0

*Note:* Outline of adverse events graded in accordance with the common terminology criteria for adverse events version 4.0. Adverse events were reported by 17 (8.06%) participants. No participants experienced life‐threatening/disabling side‐effects.

Univariable analysis showed that patients over 60 years of age (*n* = 21; 9.95%) were more likely to report AEs (OR = 10.12; 95% CI 1.38–205.73; *p* = 0.045). This finding was maintained in multivariable analysis (OR = 19.08; 95% CI 1.52–535.75; *p* = 0.036).

## 4 Discussion

These findings indicate that over a two‐year period, CBMPs were associated with statistically and clinically significant improvements in sleep quality at all timepoints up to 24 months. Benefits were most pronounced in participants with greater baseline sleep impairment. AEs were reported by fewer than 1 in 10 participants and were generally mild to moderate in severity. These results support the potential utility of CBMPs for insomnia, particularly in those with more severe baseline sleep impairment, but must be interpreted considering study limitations.

The consistent, clinically significant improvements in sleep quality demonstrated by this study support the potential therapeutic use of CBMPs for insomnia. The results provide encouraging real‐world evidence for the long‐term utility of CBMPs. This is particularly promising since current conventional therapies such as Z‐drugs and benzodiazepines have shown limited long‐term efficacy [64]. As well as this, a 2022 review by Scharner et al. [14] highlights that these medications lack high‐quality evidence to suggest improvement in sleep quality. In contrast, this study demonstrates that CBMPs may maintain effectiveness over a long period. The positive results from this study align closely with previous longitudinal studies [46, 65]. In a 2020 18‐month chart review of 37 patients with sleep disorders in Canada, 71% of participants displayed an improvement in self‐reported sleep quality [65]. While the current body of evidence is promising, most studies on CBMPs are limited by their retrospective designs, highlighting the need for more robust randomized trials to solidify findings [46, 65].

Z‐drugs and benzodiazepines act on inhibitory pathways in the central nervous system [66]. In contrast to this, CBMPs target the ECS, which has much broader implications [67, 68, 69, 70]. The broad action of CBMPs may allow them to improve sleep quality through several different physiological pathways rather than inducing sedation only. Modulating homeostasis and circadian sleep drivers could be potential mechanisms of sleep quality improvement. The therapeutic flexibility of CBMPs opens the possibility for personalized treatment using different cannabinoid formulations to elicit different effects. Notably, participants with the most severe baseline sleep impairments experienced the most meaningful improvements, suggesting that CBMPs may be particularly useful for those with treatment‐resistant insomnia symptoms. These findings support the need for further investigation into the use of CBMPs for patients who are unresponsive to conventional therapies.

Anxiety scores (GAD7) showed improvement compared to baseline at all timepoints, consistent with research linking ECS modulation to anxiolytic effects [71]. Higher THC doses at 24 months were associated with lower odds of a clinically significant improvement in GAD7, consistent with preclinical data suggesting that THC may reduce anxiety at lower doses but exacerbate it at higher doses [72]. Pilot data has also suggested that CBD may display anxiolytic effects in an inverted U‐shaped dose‐respondent manner, with moderate doses being most effective, while lower and higher doses are less effective [73]. Clinical data suggests that THC may lead to a small improvement in anxiety symptoms. However the quality of evidence supporting this finding is low [74]. THC has also been shown to induce anxiety in some individuals [75]. Interestingly, high doses of CBD have been shown to reduce THC‐induced anxiety in humans [75]. These findings further highlight the need for careful cannabinoid profiling when prescribing CBMPs.

CBMPs were generally well tolerated: 8.06% of participants reported one or more AEs, none of which were life‐threatening. Patients over 60 were more likely to experience AEs, warranting careful consideration when prescribing CBMPs to older adults. A study by Pearson et al. [76] investigated the effects of medical cannabis on older patients. In their cohort of 299 patients (mean age 66.7 ± 9.5 years), fewer than 5% reported AEs over a 6‐month period [76]. It is important to note that in contrast to this study, the majority of participants in Pearson et al.'s [76] study were female (66.22%) and cannabis naïve (90.96%), making it difficult to draw comparisons.

Insomnia was the most common AE. Insomnia has been reported as a side effect of CBMP use in previous studies [77, 78]. However, in the context of the present study, it is important to note that it was not assessed whether AEs were directly caused by CBMPs, thus it is not known whether these were related to CBMP use or secondary to fluctuations in underlying insomnia severity or other external factors. Patients may also anticipate an improvement in symptoms and therefore consider ongoing symptoms as an AE. The presence of both positive outcomes and AEs suggests a dichotomy in patient responses to CBMPs. While some patients may experience significant therapeutic benefits, others may experience little to no effect or even a worsening of symptoms, highlighting the need for further research using polysomnography.

Rising THC doses and the narrowing of PROM scores over time suggest possible tolerance from CB1 receptor downregulation [79]. This may diminish the efficacy of CBMPs over time. This study did not specifically assess dose–response relationships. Future research should explore whether tolerance develops in long‐term CBMP use and whether strategies like intermittent dosing or using CBD‐rich formulations can minimize tolerance while maintaining efficacy.

As an observational, non‐randomized study, causation cannot be inferred. Selection bias and residual confounding remain possible despite multivariable adjustment. Reliance on subjective PROMs introduces recall and reporting bias. This may result in over‐ or under‐reporting of symptoms in the context of high treatment expectancy, particularly given that participants were self‐paying for an unlicensed therapy and were therefore likely to have positive baseline expectations. Without objective sleep measures it is not possible to determine the extent to which the observed associations reflect true changes in sleep architecture rather than changes in subjective experience; the use of objective sleep measures would strengthen future studies. While multiple imputation models were used to adjust for missing data, this still may not eliminate attrition bias. While this has been shown as a robust method for handling missing data, its performance is weakers when data are missing not at random [80]. While the size of the cohort is larger than preexisting observational studies that have focused on those with a diagnosis of insomnia treated with CBMPs, the sample size is still relatively small and selective. Of note, this has affected the outcomes of the logistic regression, which have broad 95% CIs, suggesting larger cohorts are necessary to study the effects further, particularly in those with less common characteristics.

A further limitation is the multiplicity of statistical testing. In addition to the primary analysis, a large number of secondary univariable and multivariable regression models were fitted across several outcomes and predictor variables. Although the Bonferroni correction was applied within each analysis family, no correction was applied across the study as a whole, and the family‐wise Type I error rate across the full set of analyses is therefore inflated. Consequently, the secondary associations reported here, particularly those with wide confidence intervals or borderline significance, should be interpreted as hypothesis‐generating rather than confirmatory, and warrant independent replication.

CBMPs have been associated with a significant placebo response [81]. This placebo effect could be heightened because of several different factors, including media attention, previous cannabis experiences, or the psychoactive effects of CBMPs [81, 82]. In addition, paying for medications can lead to a greater placebo effect as patients may associate greater costs with higher treatment efficacy [83]. Access to CBMPs in this study was only via self‐paying private patients. Heterogeneous formulations and a predominantly male, cannabis‐experienced cohort limit generalisability. While this is the largest insomnia‐focused study of its nature so far, replication in larger, more diverse cohorts is required.

A further concern in this cohort is regression to the mean. Participants with the most severe baseline sleep impairment demonstrated the largest improvements in sleep quality at 24 months. While this is consistent with a true responder phenotype, it is also consistent with regression to the mean, whereby extreme baseline scores are partly attributable to transient factors and naturally drift toward the population mean over time, irrespective of treatment. Without an untreated control group, these two explanations cannot be separated.

The cohort was highly selected, reflecting a UK private clinic population, among which the majority had previous exposure to cannabis and were male. Insomnia is an uncommon primary indication for CBMP prescription within the UKMCR, reflecting the predominance of chronic pain and anxiety as indications in this setting. The registry does not systematically capture prior polysomnography or assessment for sleep‐disordered breathing and is therefore unable to determine the proportion of participants in whom an alternative diagnosis had been excluded. This is a meaningful limitation, given the overlap between insomnia symptoms and sleep‐disordered breathing. However, patients are not eligible to be prescribed CBMPs unless they have demonstrated a diagnosis and unsuccessful treatment with licensed medications. Consequently, it is likely the cohort does indeed represent those with a true insomnia disorder.

A further important consideration is pretreatment cannabis exposure. The majority of the cohort were current or former users of non‐prescribed cannabis at enrolment, and only 40 participants were cannabis‐naïve. Prior cannabis exposure may bias the cohort toward individuals who have previously experienced symptomatic benefit from cannabis and who therefore self‐select for treatment, potentially inflating apparent improvement; conversely, chronic prior use may attenuate response through pharmacological tolerance to THC and through carry‐over effects on baseline severity. The small cannabis‐naïve subgroup limits the statistical power of any direct comparison. An adequately powered comparison between cannabis‐naïve individuals and previous cannabis consumers is required before clinical recommendations can be made.

Over 2 years, there was an associated improvement in self‐reported sleep quality in insomnia patients treated with CBMPs. Those with severe baseline impairment showed the greatest clinical improvement. Insomnia was also the most common AE, although it is not clear whether these events were drug‐related or secondary to fluctuation in the underlying disorder. RCTs are required to assess the long‐term use of CBMPs for insomnia further to determine their true efficacy. Improvements in sleep quality in those with the poorest sleep may represent regression to the mean.

## Funding

The authors have nothing to report.

## Ethics Statement

Formal ethical approval for the UK Medical Cannabis Registry has been provided by the Health Research Authority (South West—Central Bristol Research Ethics Committee reference 22/SW/0145). Permission to Reproduce: Not applicable.

## Consent

All patients provided informed consent prior to enrolment in the UK Medical Cannabis Registry.

## Conflicts of Interest

Aran Koye is a medical student at Imperial College London. Aran Koye has no shareholdings in pharmaceutical companies. Simon Erridge is Research Director at Curaleaf Clinic. Simon Erridge is also a PhD candidate at Imperial College London. He is also a resident doctor in general practice within North‐West London, UK. Simon Erridge has no shareholdings in pharmaceutical companies. Evonne Clarke is the Patient Care Director at Curaleaf Clinic. Evonne Clarke has no shareholdings in pharmaceutical companies. Katy McLachlan is the Chief Pharmacist at Curaleaf Clinic. Katy McLachlan has no shareholdings in pharmaceutical companies. Ross Coomber is a consultant orthopedic surgeon at St George's Hospital, London, and Operations Director at Curaleaf Clinic. Ross Coomber has no shareholdings in pharmaceutical companies. James Rucker is a consultant psychiatrist at Curaleaf Clinic. James Rucker has no shareholdings in pharmaceutical companies. Mark W. Weatherall is a consultant neurologist at Curaleaf Clinic. Mark Weatherall has no shareholdings in pharmaceutical companies. Mikael H. Sodergren is a consultant hepatopancreatobiliary surgeon at Imperial College Healthcare NHS Trust, London, a clinical associate professor at Imperial College London, and the chief medical officer of Curaleaf International. Mikael Sodergren has no shareholdings in pharmaceutical companies.

## Supporting information


**Table S1:** Reported comorbidities by patients enrolled on the UK Medical Cannabis Registry and treated for insomnia.
**Table S2:** Reported indications for cannabis‐based medicinal products (CBMPs) (*n* = 211). Includes secondary and tertiary indications for the prescription of CBMPs—indicates no participants in that category.
**Table S3:** Patient‐reported outcome measures (PROMs) at baseline, 1, 3, 6, 12, 18, and 24 months (*n* = 211). A comparison of mean scores for each PROM compared to baseline, conducted with paired *t*‐tests with Bonferroni correction after repeated measures analysis of variance demonstrated statistical significance for each PROM (*p* < 0.001). The number of patients displaying minimal clinically important differences (MCID) is also shown for each timepoint after the baseline. EQ‐5D‐5L—EuroQol 5‐dimension 5‐level tool; GAD7—Generalized Anxiety Disorder‐7 score.
**Table S4:** Univariable Logistic Regression for the Association between Patient and Treatment Variables and a Minimal Clinically Important Difference (MCID) in the Single‐Item Sleep Quality Scale (SQS). BMI—body mass index; CBD—cannabidiol; CBMP—cannabis‐based medicinal product; CI—confidence interval; GAD7—Generalized Anxiety Disorder‐7; THC—(−)trans‐Δ9‐tetrahydrocannabinol.
**Table S5:** Univariable logistic regression for the association between patient and treatment variables and a minimal clinically important difference (MCID) in the generalized anxiety disorder‐7 (GAD7). BMI—body mass index; CBD—cannabidiol; CBMP—cannabis‐based medicinal product; CI—confidence interval; SQS—single‐item sleep quality scale; THC—(−)trans‐Δ9‐tetrahydrocannabinol.
**Table S6:** Multivariable logistic regression for the association between patient and treatment variables and a minimal clinically important difference (MCID) in the generalized anxiety disorder‐7 (GAD7). BMI—body mass index; CBD—cannabidiol; CBMP—cannabis‐based medicinal product; CI—confidence interval; SQS—single‐item sleep quality scale; THC—(−)trans‐Δ9‐tetrahydrocannabinol.
**Table S7:** Univariable logistic regression for the association between patient and treatment variables and an improvement the EQ‐5D‐5L Index Value. BMI—body mass index; CBD—cannabidiol; CBMP—cannabis‐based medicinal product; CI—confidence interval; GAD7—Generalized Anxiety Disorder‐7; SQS—single‐item sleep quality scale; THC—(−)trans‐Δ9‐tetrahydrocannabinol.
**Table S8:** Multivariable logistic regression for the association between patient and treatment variables and an improvement the EQ‐5D‐5L index value. BMI—body mass index; CBD—cannabidiol; CBMP—cannabis‐based medicinal product; CI—confidence interval; GAD7—Generalized Anxiety Disorder‐7; SQS—single‐item sleep quality scale; THC—(−)trans‐Δ9‐tetrahydrocannabinol.
**Table S9:** Patient global impression of change (PGIC) at all time points. IQR—interquartile rage; SD—standard deviation.

## Data Availability

The data that support the findings of this study are available on request from the corresponding author. The data are not publicly available due to privacy or ethical restrictions.
