# Supplementary material for: UK Medical Cannabis Registry: A Two‐Year Prospective Observational Cohort Study of Clinical Outcomes in Insomnia
Source: Neuropsychopharmacol Rep. 2026 Oct 26;46(4):e70180. doi: 10.1002/npr2.70180 (PMC13710906; doi:10.1002/npr2.70180)
Supplement: Supplementary file 1 [file NPR2-46-e70180-s001.docx]

**Supplementary Material**

**Table S1:** Reported comorbidities of patients enrolled on the UK Medical Cannabis Registry and treated for insomnia (n = 211).

| **Comorbidities** | **n (%)** |
| --- | --- |
| Depression or Anxiety | 100 (47.39) |
| Hypertension | 11 (5.21) |
| Arthritis | 8 (3.79) |
| Diabetes Mellitus | 8 (3.79) |
| Thyroid Dysfunction | 8 (3.79) |
| Epilepsy | 4 (1.90) |
| Liver Disease | 4 (1.90) |
| Congestive Heart Failure | 2 (0.95) |
| Solid Tumour | 2 (0.95) |
| Cerebrovascular Accident/Transient Ischaemic Attack | 1 (0.47) |
| Hemiplegia | 1 (0.47) |
| Moderate to Severe Kidney Disease | 1 (0.47) |
| Myocardial Infarction | 1 (0.47) |
| Peptic Ulcer Disease | 1 (0.47) |
| Peripheral Vascular Disease | 1 (0.47) |
| Acquired Immunodeficiency Syndrome | 0 (0.00) |
| Chronic Obstructive Pulmonary Disease | 0 (0.00) |
| Connective Tissue Disease | 0 (0.00) |
| Dementia | 0 (0.00) |
| Leukaemia | 0 (0.00) |
| Lymphoma | 0 (0.00) |
| Venous Thromboembolism | 0 (0.00) |

**Table S2: Reported indications for cannabis-based medicinal products (CBMPs) (n = 211).** Includes secondary and tertiary indications for the prescription of CBMPs. *— indicates no participants in that category.*

| **Indication** | **Primary, n (%)** | **Secondary, n (%)** | **Tertiary, n (%)** |
| --- | --- | --- | --- |
| **Insomnia** | 211 (100.00) | — | — |
| **Anxiety** | — | 38 (18.01) | 2 (0.95) |
| **Chronic Non-Cancer Pain** | — | 12 (5.69) | 1 (0.47) |
| **Depression** | — | 7 (3.32) | 8 (3.79) |
| **Migraine** | — | 6 (2.84) | 1 (0.47) |
| **Post-Traumatic Stress Disorder** | — | 2 (0.95) | 1 (0.47) |
| **Agoraphobia** | — | 1 (0.47) | 0 (0.00) |
| **Attention Deficit Hyperactivity Disorder** | — | 1 (0.47) | 2 (0.95) |
| **Autism Spectrum Disorder** | — | 1 (0.47) | 1 (0.47) |
| **Eating Disorder** | — | 0 (0.00) | 1 (0.47) |
| **Epilepsy (adult)** | — | 1 (0.47) | 1 (0.47) |
| **Fibromyalgia** | — | 0 (0.00) | 1 (0.47) |
| **Headache** | — | 1 (0.47) | 0 (0.00) |
| **Neuropathic Pain** | — | 1 (0.47) | 0 (0.00) |
| **Parkinson's disease** | — | 1 (0.47) | 0 (0.00) |
| **Social Phobia** | — | 0 (0.00) | 1 (0.47) |

**Table S3: Patient-reported outcome measures (PROMs) at baseline and at 1, 3, 6, 12, 18, and 24 months (n = 211). Mean scores at each follow-up timepoint were compared with baseline using paired t-tests with Bonferroni correction following repeated-measures analysis of variance. All comparisons were statistically significant (p < 0.001) except the EQ-5D-5L index value at 24 months (p > 0.999). The number of patients achieving the minimal clinically important difference (MCID) is shown for each timepoint after baseline. *EQ-5D-5L, EuroQol 5-dimension 5-level; GAD-7, Generalised Anxiety Disorder-7; MCID, minimal clinically important difference; PROM, patient-reported outcome measure; SD, standard deviation; SQS, Single-Item Sleep Quality Scale.***

***There is no established MCID for the EQ-5D-5L. This column therefore shows the number of patients with any improvement in the EQ-5D-5L index value.**

| **Measure** | **Timepoint** | **Mean (± SD)** | **p** | **Patients achieving MCID, n (%)*** |
| --- | --- | --- | --- | --- |
| **SQS** | **Baseline** | 2.46 (± 2.21) | — | — |
|  | 1 Month | 5.79 (± 2.55) | p < 0.001 | 129 (61.14%) |
|  | 3 Months | 5.91 (± 2.31) | p < 0.001 | 129 (61.14%) |
|  | 6 Months | 5.75 (± 2.28) | p < 0.001 | 125 (59.24%) |
|  | 12 Months | 6.23 (± 2.36) | p < 0.001 | 138 (65.40%) |
|  | 18 Months | 6.17 (± 2.42) | p < 0.001 | 133 (63.03%) |
|  | 24 Months | 5.14 (± 2.75) | p < 0.001 | 106 (50.24%) |
| **GAD-7** | **Baseline** | 10.41 (± 6.08) | — | — |
|  | 1 Month | 5.31 (± 4.65) | p < 0.001 | 122 (57.82%) |
|  | 3 Months | 5.27 (± 4.95) | p < 0.001 | 115 (55.83%) |
|  | 6 Months | 5.26 (± 4.52) | p < 0.001 | 116 (54.98%) |
|  | 12 Months | 4.69 (± 4.24) | p < 0.001 | 121 (63.21%) |
|  | 18 Months | 5.99 (± 5.38) | p < 0.001 | 123 (58.29%) |
|  | 24 Months | 7.36 (± 5.91) | p < 0.001 | 103 (48.82%) |
| **EQ-5D-5L Index Value** | **Baseline** | 0.63 (± 0.28) | — | — |
|  | 1 Month | 0.76 (± 0.19) | p < 0.001 | 137 (64.02%) |
|  | 3 Months | 0.75 (± 0.20) | p < 0.001 | 135 (68.01%) |
|  | 6 Months | 0.78 (± 0.18) | p < 0.001 | 148 (69.73%) |
|  | 12 Months | 0.73 (± 0.20) | p < 0.001 | 118 (57.28%) |
|  | 18 Months | 0.73 (± 0.20) | p < 0.001 | 123 (58.29%) |
|  | 24 Months | 0.64 (± 0.25) | p > 0.999 | 107 (50.71%) |

**Table S4: Univariable logistic regression for the association between patient and treatment variables and a minimal clinically important difference (MCID) in the Single-Item Sleep Quality Scale (SQS).** *BMI, body mass index; CBD, cannabidiol; CBMP, cannabis-based medicinal product; CI, confidence interval; GAD-7, Generalised Anxiety Disorder-7; Q1, first quartile; Q3, third quartile; THC, (−)-trans-Δ9-tetrahydrocannabinol. — denotes the reference category.*

| **Category** | **n** | **Odds ratio (95% CI)** | **p** |
| --- | --- | --- | --- |
| **Age (years)** |  |  |  |
| ≤30 | 43 | — | — |
| 31–40 | 62 | 1.17 (0.54–2.54) | 0.693 |
| 41–50 | 54 | 1.02 (0.46–2.26) | 0.967 |
| 51–60 | 30 | 1.64 (0.65–4.28) | 0.301 |
| >60 | 21 | 0.82 (0.28–2.33) | 0.713 |
| **Sex** |  |  |  |
| Male | 149 | — | — |
| Female | 58 | 1.31 (0.72–2.42) | 0.378 |
| **BMI (kg/m²)** |  |  |  |
| 20–24.99 | 69 | — | — |
| <20 | 15 | 1.38 (0.45–4.50) | 0.583 |
| 25–29.99 | 72 | 0.92 (0.47–1.78) | 0.796 |
| 30–34.99 | 33 | 0.60 (0.25–1.37) | 0.229 |
| ≥35 | 14 | 1.22 (0.38–4.06) | 0.734 |
| **Cannabis Status** |  |  |  |
| Never Used | 37 | — | — |
| Current User | 126 | 0.61 (0.29–1.25) | 0.178 |
| Ex-user | 45 | 1.01 (0.43–2.40) | 0.979 |
| **CBMP Route of Administration at 24 months** |  |  |  |
| Oil | 33 | — | — |
| Dried Flower & Oil | 68 | 0.84 (0.37–1.90) | 0.680 |
| Dried Flower | 87 | 0.96 (0.44–2.09) | 0.915 |
| Other | 20 | 0.73 (0.24–2.19) | 0.577 |
| **Total CBD dose (mg/day) at 24 months** |  |  |  |
| Minimum to Q1 | 51 | — | — |
| Q1 to Median | 54 | 1.21 (0.57–2.61) | 0.622 |
| Median to Q3 | 51 | 1.59 (0.73–3.47) | 0.242 |
| Q3 to Maximum | 53 | 1.58 (0.73–3.42) | 0.245 |
| **Total THC dose (mg/day) at 24 months** |  |  |  |
| Minimum to Q1 | 64 | — | — |
| Q1 to Median | 41 | 0.56 (0.25–1.23) | 0.150 |
| Median to Q3 | 50 | 0.44 (0.21–0.92) | 0.032 |
| Q3 to Maximum | 53 | 0.94 (0.45–1.95) | 0.860 |
| **Baseline SQS** |  |  |  |
| 4–10 | 41 | — | — |
| 1–3 | 129 | 4.59 (2.06–11.36) | <0.001 |
| 0 | 40 | 14.64 (5.28–45.46) | <0.001 |
| **Baseline GAD-7** |  |  |  |
| <5 | 43 | — | — |
| 5–9 | 50 | 1.57 (0.69–3.67) | 0.288 |
| 10–14 | 56 | 2.48 (1.11–5.70) | 0.029 |
| ≥15 | 60 | 3.45 (1.56–7.95) | 0.003 |

**Table S5: Univariable logistic regression for the association between patient and treatment variables and a minimal clinically important difference (MCID) in the Generalised Anxiety Disorder-7 (GAD-7).** *BMI, body mass index; CBD, cannabidiol; CBMP, cannabis-based medicinal product; CI, confidence interval; Q1, first quartile; Q3, third quartile; SQS, Single-Item Sleep Quality Scale; THC, (−)-trans-Δ9-tetrahydrocannabinol. — denotes the reference category.*

| **Category** | **n** | **Odds ratio (95% CI)** | **p** |
| --- | --- | --- | --- |
| **Age (years)** |  |  |  |
| ≤30 | 43 | — | — |
| 31–40 | 62 | 0.59 (0.27–1.27) | 0.180 |
| 41–50 | 54 | 0.82 (0.37–1.82) | 0.624 |
| 51–60 | 30 | 0.58 (0.22–1.47) | 0.256 |
| >60 | 21 | 0.69 (0.24–1.97) | 0.487 |
| **Sex** |  |  |  |
| Male | 149 | - | - |
| Female | 58 | 0.73 (0.39–1.34) | 0.308 |
| **BMI (kg/m²)** |  |  |  |
| 20–24.99 | 69 | — | — |
| <20 | 15 | 0.85 (0.27–2.62) | 0.776 |
| 25–29.99 | 72 | 0.78 (0.40–1.51) | 0.456 |
| 30–34.99 | 33 | 0.91 (0.40–2.10) | 0.832 |
| ≥35 | 14 | 1.30 (0.41–4.31) | 0.662 |
| **Cannabis Status** |  |  |  |
| Never Used | 37 | - | - |
| Current User | 126 | 1.00 (0.49–2.06) | 0.990 |
| Ex-user | 45 | 1.26 (0.54–2.99) | 0.592 |
| **CBMP Route of Administration at 24 months** |  |  |  |
| Oil | 33 | - | - |
| Dried Flower & Oil | 68 | 1.92 (0.85–4.41) | 0.118 |
| Dried Flower | 87 | 0.72 (0.33–1.58) | 0.405 |
| Other | 20 | 0.91 (0.30–2.74) | 0.873 |
| **Total CBD dose (mg/day) at 24 months** |  |  |  |
| Minimum to Q1 | 51 | — | — |
| Q1 to Median | 54 | 1.65 (0.77–3.60) | 0.202 |
| Median to Q3 | 51 | 2.56 (1.17–5.72) | 0.020 |
| Q3 to Maximum | 53 | 1.47 (0.68–3.22) | 0.327 |
| **Total THC dose (mg/day) at 24 months** |  |  |  |
| Minimum to Q1 | 64 | — | — |
| Q1 to Median | 41 | 1.17 (0.54–2.57) | 0.696 |
| Median to Q3 | 50 | 0.56 (0.26–1.17) | 0.128 |
| Q3 to Maximum | 53 | 0.88 (0.43–1.81) | 0.729 |
| **Baseline SQS** |  |  |  |
| 4–10 | 41 | — | — |
| 1–3 | 129 | 2.95 (1.40–6.61) | 0.006 |
| 0 | 40 | 5.23 (2.08–13.96) | <0.001 |
| **Baseline GAD-7** |  |  |  |
| <5 | 43 | — | — |
| 5–9 | 50 | 31.86 (6.14–586.78) | <0.001 |
| 10–14 | 56 | 79.20 (15.42–1456.26) | <0.001 |
| ≥15 | 60 | 132.00 (25.37–2440.46) | <0.001 |

**Table S6: Multivariable logistic regression for the association between patient and treatment variables and a minimal clinically important difference (MCID) in the Generalised Anxiety Disorder-7 (GAD-7). Complete-case analysis (n = 203). All variables shown were entered simultaneously.** *BMI, body mass index; CBD, cannabidiol; CBMP, cannabis-based medicinal product; CI, confidence interval; Q1, first quartile; Q3, third quartile; SQS, Single-Item Sleep Quality Scale; THC, (−)-trans-Δ9-tetrahydrocannabinol. — denotes the reference category.*

| **Category** | **n** | **Adjusted odds ratio (95% CI)** | **p** |
| --- | --- | --- | --- |
| **Age (years)** |  |  |  |
| ≤30 | 43 | — | — |
| 31–40 | 59 | 0.85 (0.28–2.56) | 0.773 |
| 41–50 | 52 | 0.81 (0.27–2.37) | 0.698 |
| 51–60 | 29 | 0.54 (0.14–1.98) | 0.350 |
| >60 | 20 | 0.54 (0.10–2.96) | 0.473 |
| **Sex** |  |  |  |
| Male | 149 | — | — |
| Female | 54 | 0.31 (0.12–0.73) | 0.010 |
| **BMI (kg/m²)** |  |  |  |
| 20–24.99 | 69 | — | — |
| <20 | 15 | 0.84 (0.19–3.74) | 0.814 |
| 25–29.99 | 72 | 0.97 (0.38–2.47) | 0.944 |
| 30–34.99 | 33 | 0.91 (0.30–2.76) | 0.870 |
| ≥35 | 14 | 1.60 (0.34–8.54) | 0.566 |
| **Cannabis Status** |  |  |  |
| Never Used | 37 | — | — |
| Current User | 122 | 0.52 (0.13–2.02) | 0.348 |
| Ex-user | 44 | 0.61 (0.14–2.54) | 0.495 |
| **CBMP Route of Administration at 24 months** |  |  |  |
| Oil | 33 | — | — |
| Dried Flower & Oil | 64 | 8.44 (1.33–66.38) | 0.030 |
| Dried Flower | 86 | 2.66 (0.45–19.20) | 0.299 |
| Other | 20 | 1.88 (0.29–14.54) | 0.518 |
| **Total CBD dose (mg/day) at 24 months** |  |  |  |
| Minimum to Q1 | 51 | — | — |
| Q1 to Median | 51 | 1.73 (0.47–6.61) | 0.413 |
| Median to Q3 | 49 | 1.53 (0.37–6.53) | 0.561 |
| Q3 to Maximum | 52 | 1.44 (0.38–5.48) | 0.591 |
| **Total THC dose (mg/day) at 24 months** |  |  |  |
| Minimum to Q1 | 64 | — | — |
| Q1 to Median | 37 | 0.09 (0.01–0.45) | 0.006 |
| Median to Q3 | 50 | 0.08 (0.01–0.40) | 0.004 |
| Q3 to Maximum | 52 | 0.13 (0.02–0.68) | 0.023 |
| **Baseline SQS** |  |  |  |
| 4–10 | 41 | — | — |
| 1–3 | 124 | 1.08 (0.36–3.25) | 0.896 |
| 0 | 38 | 1.55 (0.42–5.90) | 0.513 |
| **Baseline GAD-7** |  |  |  |
| <5 | 43 | — | — |
| 5–9 | 49 | 73.92 (10.58–1643.84) | <0.001 |
| 10–14 | 54 | 182.94 (25.32–4149.22) | <0.001 |
| ≥15 | 57 | 343.92 (44.62–8122.07) | <0.001 |

**Table S7: Univariable logistic regression for the association between patient and treatment variables and an improvement in the EuroQol 5-dimension 5-level (EQ-5D-5L) index value.** *BMI, body mass index; CBD, cannabidiol; CBMP, cannabis-based medicinal product; CI, confidence interval; GAD-7, Generalised Anxiety Disorder-7; Q1, first quartile; Q3, third quartile; SQS, Single-Item Sleep Quality Scale; THC, (−)-trans-Δ9-tetrahydrocannabinol. — denotes the reference category.*

| **Category** | **n** | **Odds ratio (95% CI)** | **p** |
| --- | --- | --- | --- |
| **Age (years)** |  |  |  |
| ≤30 | 43 | — | — |
| 31–40 | 62 | 0.99 (0.45–2.16) | 0.976 |
| 41–50 | 54 | 1.39 (0.63–3.12) | 0.417 |
| 51–60 | 30 | 1.80 (0.71–4.70) | 0.221 |
| >60 | 21 | 1.60 (0.56–4.67) | 0.380 |
| **Sex** |  |  |  |
| Male | 149 | — | — |
| Female | 58 | 1.16 (0.64–2.14) | 0.624 |
| **BMI (kg/m²)** |  |  |  |
| 20–24.99 | 69 | — | — |
| <20 | 15 | 1.11 (0.36–3.49) | 0.855 |
| 25–29.99 | 72 | 1.09 (0.56–2.11) | 0.807 |
| 30–34.99 | 33 | 0.91 (0.40–2.10) | 0.832 |
| ≥35 | 14 | 0.97 (0.30–3.12) | 0.961 |
| **Cannabis Status** |  |  |  |
| Never Used | 37 | — | — |
| Current User | 126 | 1.00 (0.49–2.04) | 1.000 |
| Ex-user | 45 | 1.14 (0.49–2.69) | 0.759 |
| **CBMP Route of Administration at 24 months** |  |  |  |
| Oil | 33 | - | — |
| Dried Flower & Oil | 68 | 0.80 (0.35–1.80) | 0.590 |
| Dried Flower | 87 | 0.78 (0.35–1.70) | 0.536 |
| Other | 20 | 0.80 (0.26–2.41) | 0.690 |
| **Total CBD dose (mg/day) at 24 months** |  |  |  |
| Minimum to Q1 | 51 | — | — |
| Q1 to Median | 54 | 1.78 (0.83–3.88) | 0.144 |
| Median to Q3 | 51 | 2.56 (1.17–5.72) | 0.020 |
| Q3 to Maximum | 53 | 1.85 (0.86–4.05) | 0.120 |
| **Total THC dose (mg/day) at 24 months** |  |  |  |
| Minimum to Q1 | 64 | — | — |
| Q1 to Median | 41 | 1.48 (0.68–3.27) | 0.322 |
| Median to Q3 | 50 | 0.91 (0.43–1.91) | 0.807 |
| Q3 to Maximum | 53 | 1.64 (0.79–3.41) | 0.185 |
| **Baseline SQS** |  |  |  |
| 4–10 | 41 | — | — |
| 1–3 | 129 | 1.65 (0.82–3.42) | 0.168 |
| 0 | 40 | 3.02 (1.24–7.59) | 0.016 |
| **Baseline GAD-7** |  |  |  |
| <5 | 43 | — | — |
| 5–9 | 50 | 2.34 (1.00–5.69) | 0.053 |
| 10–14 | 56 | 3.67 (1.60–8.79) | 0.003 |
| ≥15 | 60 | 5.50 (2.40–13.29) | <0.001 |

**Table S8: Multivariable logistic regression for the association between patient and treatment variables and an improvement in the EuroQol 5-dimension 5-level (EQ-5D-5L) index value. Complete-case analysis (n = 203). All variables shown were entered simultaneously.** *BMI, body mass index; CBD, cannabidiol; CBMP, cannabis-based medicinal product; CI, confidence interval; GAD-7, Generalised Anxiety Disorder-7; Q1, first quartile; Q3, third quartile; SQS, Single-Item Sleep Quality Scale; THC, (−)-trans-Δ9-tetrahydrocannabinol. — denotes the reference category.*

| **Category** | **n** | **Adjusted odds ratio (95% CI)** | **p** |
| --- | --- | --- | --- |
| **Age (years)** |  |  |  |
| ≤30 | 43 | — | — |
| 31–40 | 59 | 1.30 (0.53–3.18) | 0.568 |
| 41–50 | 52 | 1.49 (0.60–3.77) | 0.391 |
| 51–60 | 29 | 2.34 (0.77–7.42) | 0.138 |
| >60 | 20 | 2.01 (0.56–7.50) | 0.290 |
| **Sex** |  |  |  |
| Male | 149 | — | — |
| Female | 54 | 0.86 (0.41–1.80) | 0.689 |
| **BMI (kg/m²)** |  |  |  |
| 20–24.99 | 69 | — | — |
| <20 | 15 | 1.27 (0.36–4.63) | 0.709 |
| 25–29.99 | 72 | 1.43 (0.67–3.10) | 0.356 |
| 30–34.99 | 33 | 0.88 (0.34–2.26) | 0.795 |
| ≥35 | 14 | 0.93 (0.25–3.51) | 0.911 |
| **Cannabis Status** |  |  |  |
| Never Used | 37 | — | — |
| Current User | 122 | 1.14 (0.41–3.22) | 0.795 |
| Ex-user | 44 | 1.18 (0.38–3.74) | 0.770 |
| **CBMP Route of Administration at 24 months** |  |  |  |
| Oil | 33 | — | - |
| Dried Flower & Oil | 64 | 0.35 (0.09–1.31) | 0.122 |
| Dried Flower | 86 | 0.58 (0.16–2.02) | 0.390 |
| Other | 20 | 0.33 (0.07–1.41) | 0.137 |
| **Total CBD dose (mg/day) at 24 months** |  |  |  |
| Minimum to Q1 | 51 | — | — |
| Q1 to Median | 51 | 1.52 (0.55–4.24) | 0.416 |
| Median to Q3 | 49 | 1.93 (0.63–6.05) | 0.251 |
| Q3 to Maximum | 52 | 1.46 (0.50–4.25) | 0.487 |
| **Total THC dose (mg/day) at 24 months** |  |  |  |
| Minimum to Q1 | 64 | — | — |
| Q1 to Median | 37 | 2.43 (0.74–8.30) | 0.148 |
| Median to Q3 | 50 | 1.20 (0.38–3.83) | 0.756 |
| Q3 to Maximum | 52 | 2.01 (0.60–6.91) | 0.260 |
| **Baseline SQS** |  |  |  |
| 4–10 | 41 | - | - |
| 1–3 | 124 | 1.01 (0.43–2.34) | 0.985 |
| 0 | 38 | 1.67 (0.57–4.96) | 0.352 |
| **Baseline GAD-7** |  |  |  |
| <5 | 43 | — | — |
| 5–9 | 49 | 2.01 (0.78–5.36) | 0.152 |
| 10–14 | 54 | 3.24 (1.23–8.98) | 0.020 |
| ≥15 | 57 | 4.51 (1.64–13.08) | 0.004 |

**Table S9: Patient Global Impression of Change (PGIC) at all timepoints.** *IQR, interquartile range; SD, standard deviation.*

| **Timepoint** | **Mean PGIC (± SD)** | **Median PGIC (IQR)** |
| --- | --- | --- |
| 1 month | 5.42 (± 1.50) | 6 (5–6) |
| 3 months | 5.84 (± 1.10) | 6 (5–7) |
| 6 months | 5.82 (± 1.01) | 6 (5–6) |
| 12 months | 5.72 (± 1.28) | 6 (5–7) |
| 18 months | 5.82 (± 1.13) | 6 (5–7) |
| 24 months | 5.59 (± 1.44) | 6 (5–7) |
